# A new mode of inhibition

**DOI:** 10.7554/eLife.101446

**Published:** 2024-08-19

**Authors:** Andrew D Huber, Taosheng Chen

**Affiliations:** 1 Department of Chemical Biology and Therapeutics, St. Jude Children’s Research Hospital Memphis United States

**Keywords:** nuclear receptors, PPARγ, structural biology, antagonists

## Abstract

Complementary structural biology approaches reveal how an agonist and a covalent inhibitor simultaneously bind to a nuclear receptor.

**Related research article** Shang J, Kojetin DJ. 2024. Unanticipated mechanisms of covalent inhibitor and synthetic ligand cobinding to PPARγ. *eLife* **13**:RP99782. doi: 10.7554/eLife.99782.

Shuttling between a cell’s cytoplasm and nucleus are nuclear receptor proteins, which are involved in a diverse number of physiological and pathological roles. These nuclear receptors can be activated by a range of ligands – such as vitamins, hormones and fatty acids – which trigger the receptor to upregulate the transcription of certain genes (Nagy and Schwabe, 2004). Due to their broad biological functions, nuclear receptors are a major drug target in pharmacology (Santos et al., 2017). These medicines can either activate the receptor (agonists), inhibit receptor activity (inverse agonists), or occupy the ligand-binding pocket so that other molecules cannot access it (antagonists).

One such nuclear receptor, peroxisome proliferator-activated receptor gamma (or PPARγ for short), regulates genes involved in producing fatty tissue and controlling blood glucose levels. Various ligands can manipulate PPARγ activity, including the body’s endogenous lipids and fatty acids, as well as anti-diabetic drugs, such as rosiglitazone. Although rosiglitazone and its related synthetic compounds have been approved by the Federal Drug Administration (FDA) in the United States, their global use has been limited due to the adverse effects they can cause (Kroker and Bruning, 2015). As a result, there is growing interest in finding other drugs that can target PPARγ. However, to achieve this, researchers need a better understanding of how molecules interact with PPARγ and modulate its activity, including compounds used in basic scientific studies, as these experiments will inform the development of pharmaceutical drugs.

Two covalent inhibitors, called GW9662 and T0070907, are commonly used in the laboratory to study the potency of synthetic ligands for the PPARγ receptor. Initially, these two compounds were thought to be antagonists that block the activity of PPARγ by occupying the ligand-binding site to prevent agonists binding. However, previous studies found that these covalent inhibitors do not stop all agonists from activating PPARγ (Hughes et al., 2014). In addition, it has been shown that the ligand-binding pocket of PPARγ can bind to multiple ligands at a time, including co-binding with an agonist and an antagonist simultaneously (Shang et al., 2018). Now, in eLife, Jinsai Shang (Scripps Research and Guangzhou Medical University) and Douglas Kojetin (Scripps and Vanderbilt University) report new insights into the inhibitory properties of GW9662 and T0070907 (Shang and Kojetin, 2024).

First, Shang and Kojetin profiled how co-activators and co-repressor proteins – which help to promote and inhibit gene expression, respectively – are recruited to PPARγ when GW9662 or T0070907 are present. Typically, agonists make it easier for co-activators but harder for co-repressors to bind to nuclear receptors, while inverse agonists have the opposite effect, and antagonists are somewhere between the two. Shang and Kojetin found that some agonists could still induce PPARγ to interact with co-activators, even when its ligand-binding pocket was also covalently bound to GW9662 or T0070907. This suggests that neither GW9662-bound PPARγ or T0070907-bound PPARγ is fully inhibited.

This finding was corroborated by studying the structure of PPARγ using NMR spectroscopy, which revealed that agonists could induce an active PPARγ conformation even when GW9662 or T0070907 was present. To investigate how this happens, Shang and Kojetin solved crystal structures of the ligand-binding pocket of PPARγ that were covalently bound to GW9662 or T0070907 and had been subsequently incubated with an agonist. Surprisingly, they found that agonists only slightly adjusted their binding modes when GW9662 or T0070907 were present. Instead, it was the covalent inhibitors that underwent pronounced conformational changes, which allowed agonists to be incorporated into the ligand-binding pocket (Figure 1).

**Figure 1.**
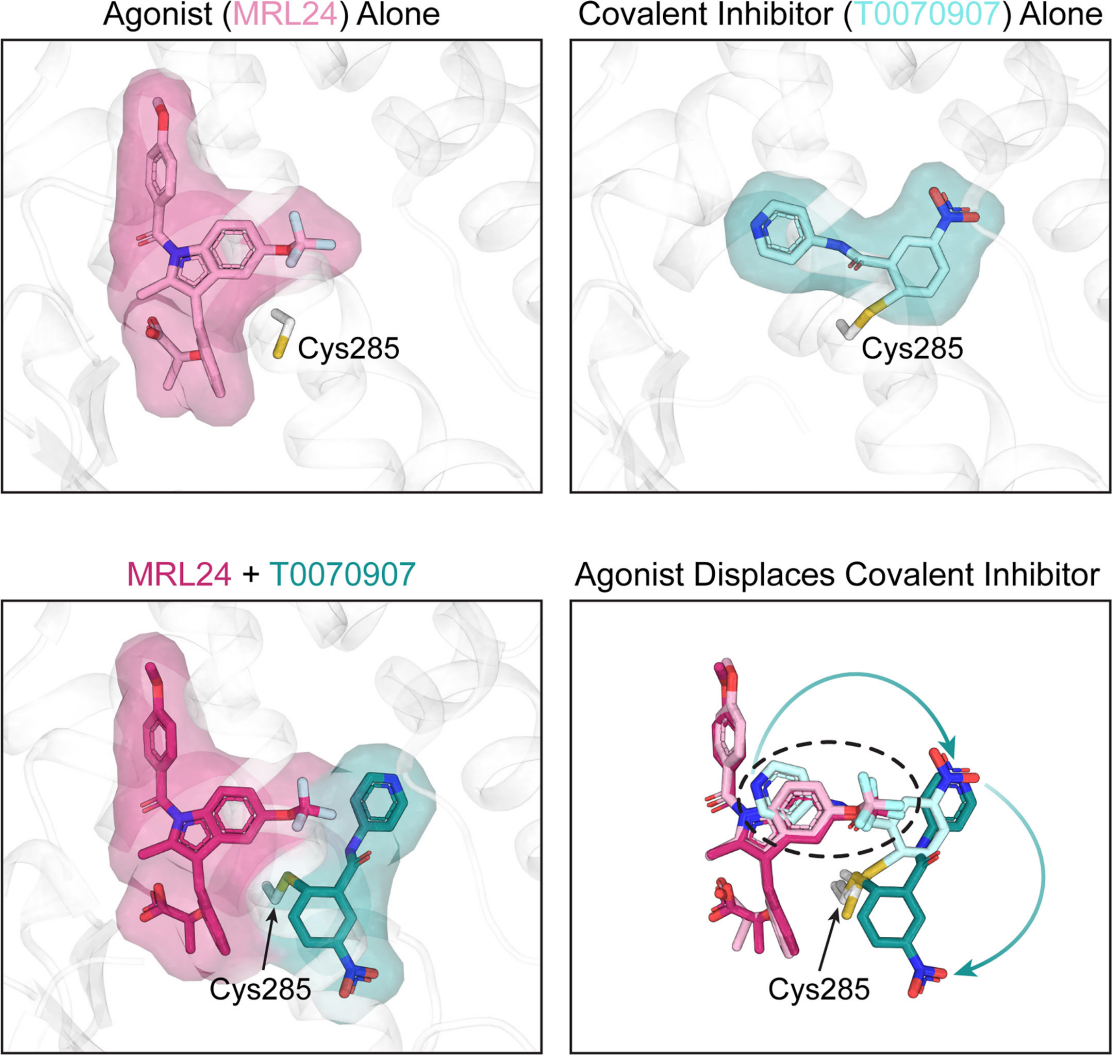
Crystal structures of the PPARγ ligand-binding pocket. When an agonist or a covalent inhibitor binds to PPARγ, it alters the structure of the ligand-binding pocket in PPARγ. The top left image shows the crystal structure of the PPARγ ligand-binding pocket (white) when bound to an agonist called MRL24 (light pink). The top right image shows the crystal structure of the PPARγ ligand-binding pocket when bound to T0070907 (light cyan), a covalent inhibitor that attaches via the amino acid Cys285. When MRL24 and T0070907 simultaneously bind to PPARγ, T0070907 is displaced (bottom left). The site where MRL24 sits in the PPARγ ligand-binding pocket overlaps with the site that T0070907 occupies (bottom right, black dashed ring). This clash causes the agonist (ML24) to displace the covalent inhibitor (T0070907) and shift it to a different position.

Image credit: Andrew D Huber; structure of MRL24 alone (PDB ID 2Q5P), T0070907 alone (PDB ID 6C1I), and MRL24/T0070907 together (Shang and Kojetin, 2024).

The classical model of receptor antagonism is simple: an agonist cannot bind to a pocket that is plugged by an antagonist. However, the findings of Shang and Kojetin suggest that the commonly used covalent inhibitors GW9662 and T0070907 act by a different mechanism. The covalent inhibitor favors an inactive PPARγ state when attached to the receptor. However, rather than preventing agonists from binding to the receptor, these compounds allow agonists to bind alongside the inhibitor, which leads to an inhibitor/agonist co-bound state. As higher amounts of agonists are added to the system, the inhibitor/agonist co-bound state may become more dominant and lead to PPARγ activation.

These findings provide a structural explanation for why GW9662 and T0070907 do not block the activity of all PPARγ agonists (Hughes et al., 2014). They also demonstrate that PPARγ antagonists that are completely inhibitory are needed to fully understand the biology of this nuclear receptor. More generally, the results demonstrate why it is important to fully characterize how ligands bind to receptors before using them as tools for studying biological functions.
